# Inhaled iloprost improves gas exchange in patients with COVID-19 and acute respiratory distress syndrome

**DOI:** 10.1186/s13054-021-03690-7

**Published:** 2021-07-21

**Authors:** Natalia A. Tsareva, Sergey N. Avdeev, Djuro Kosanovic, Ralph Theo Schermuly, Natalia V. Trushenko, Galina V. Nekludova

**Affiliations:** 1 Department of Pulmonology, I.M. Sechenov First Moscow State Medical University (Sechenov University), Healthcare Ministry of Russia, Trubetskaya Street 8, Moscow, Russia 119991; 2 Department of Internal Medicine, Justus-Liebig University Giessen, Member of the German Center for Lung Research (DZL), Giessen, Germany

**Keywords:** Iloprost, Inhalation, SARS-CoV-2, COVID-19, Acute respiratory distress syndrome

*To the Editor,*

Severe acute respiratory syndrome coronavirus (SARS-CoV)-2 outbreak that began in 2019 and spread rapidly across the world has been demonstrated to cause viral pneumonia, acute respiratory distress syndrome (ARDS) and multi-organ system failure [1]. Given the lack of scientific data, efforts are focused on an empirical search for therapeutic strategies to ensure the adequate gas exchange, including methods that can be applied in intensive care unit (ICU) setting. Iloprost is a synthetic analogue of prostacyclin and recent studies investigated its efficacy when applied via infusion in the context of COVID-19 [2, 3]. In addition, inhaled iloprost is a well-known option for the treatment of pulmonary hypertension (PH) [4]. Therefore, in the current study we have analyzed the effects of inhaled iloprost on gas exchange in patients with COVID-19 associated ARDS.

This case–control study was conducted in the Pulmonology Department of university-affiliated hospital (Sechenov University) between April 8, 2020, and May 20, 2020. The study was approved by the local ethics committee of Sechenov University, and written informed consent was obtained from all patients. Eligible patients were subjects aged over 18 years with SARS-CoV-2 infection confirmed by real-time PCR and ARDS according to the Berlin definition [5] and PaO_2_/FiO_2_ ≤ 200 mmHg. The exclusion criteria considered need for immediate endotracheal intubation and unstable hemodynamics. The primary objective was to assess the effect of inhaled iloprost on PaO_2_/FiO_2_ in patients with ARDS on Day 5. Iloprost was administered with a vibrating mesh nebulizer (Aeroneb Solo; Aerogen) four times per day (20 μg per administration) for 5 days. The control patients were selected based on the same enrollment criteria and we have prospectively recorded the measured parameters on the same data chart. The matching of the controls and patients treated with iloprost was performed based on the following criteria: age (within ± 5 years); National Early Warning Score (NEWS)-2 score on admission (within ± 1 points) and PaO_2_/FiO_2_ on admission (within ± 20 mmHg). Computed tomography (CT) scan was performed and CT severity score was calculated as 5-point scale according to the degree of lung involvement: (0) no involvement, (1) less than 25%, (2) 25–50%, (3) 50–75% and (4) more than 75% [6]. All adverse events (AE) and serious AE possibly related to inhaled iloprost were documented.

Twenty-three consecutive patients received at least one iloprost inhalation and 22 patients were included into the control group. The baseline demographic, clinical and laboratory characteristics did not differ significantly between the groups (Table 1). Time between the symptom onset and iloprost administration was 8.0 ± 0.5 days. On day 5, iloprost therapy led to the significant improvement in SpO_2_/FiO_2_ and PaO_2_/FiO_2_ compared to the baseline and controls (Fig. 1). There was also a significant reduction of the Borg dyspnea score (6 vs. 4, *p* = 0.01). Three patients in iloprost group and 6 patients in control group were transferred to ICU due to rapidly progressive respiratory failure. Remaining patients were free of supplemental oxygen/continuous positive airway pressure at the end of follow-up. The overall iloprost safety profile was similar to that observed in previous studies. The most common AE were flushing (*n* = 5; 21.7%) and jaw pain (*n* = 3; 13.0%). There were no cases of AE-related iloprost discontinuation.

**Table 1:** Baseline characteristics of the study population

	Iloprost (*n* = 23)	Control (*n* = 22)
Demographic variables		
Age, years	62 (53–68)	60 (54–69)
Male, *n* (%)	15 (65.2)	17 (77.3)
Caucasian, *n* (%)	23 (100)	22 (100)
Anthropometric measures and risk factors		
Smokers, n (%)	8 (34.8)	10 (45.5)
BMI, kg/m^2^	31.0 (28.0–34.8)	32.0 (26.5–39.6)
Medical history		
Cardiovascular disease, *n* (%)	8 (34.8)	9 (40.9)
Chronic lung disease, *n* (%)	0 (0)	1 (4.5)
Diabetes mellitus, *n* (%)	6 (26.1)	7 (31.8)
Chronic kidney disease, *n* (%)	3 (13.0)	1 (4.5)
Clinical variables		
Cough, *n* (%)	21 (91.3)	21 (95.4)
Dyspnea, *n* (%)	21 (91.3)	19 (86.4)
Fever, *n* (%)	19 (82.6)	18 (81.8)
Borg dyspnea scale	6 (5–8)	5 (2–8)
Laboratory tests		
WBC, 10^9^/L	5.9 (5.1–8.8)	6.8 (5.2–8.3)
C-reactive protein, mg/L	131 (102–190)	128 (89–186)
D-dimer, µg/mL	2.9 (1.9–3.8)	3.5 (1.9–4.6)
Blood gases		
PaO_2_, mmHg	65.8 (55.1–78.1)	62.0 (49.0–77.7)
PaCO_2_, mmHg	32.0 (29.2–35.0)	28.8 (23.8–32.7)
SpO_2_, %	89 (88–90)	90 (87–93)
PaO_2_/FiO_2_, mmHg	131 (120–138)	130 (114–168)
Computed tomography		
CT severity scale, 0/1/2/3/4, *n* (%)	0 (0)/0 (0)/7 (30.4)/9 (39.1)/7 (30.4)	0 (0)/0 (0)/5 (22.7)/14 (63.6)/3 (13.6)
Medications		
Vasopressors, *n* (%)	0 (0)	0 (0)
Corticosteroids, *n* (%)	15 (65.2)	17 (77.3)
Hydroxychloroquine, *n* (%)	21 (91.3)	19 (86.4)
Azithromycin, *n* (%)	21 (91.3)	19 (86.4)
Respiratory support		
Supplemental oxygen, *n* (%)	16 (69.6)	14 (63.6)
CPAP, *n* (%)	7 (30.4)	8 (36.4)

Data are expressed as absolute values (%) or median (interquartile range)

*BMI* body mass index, *WBC* white blood cells, *PaO*_*2*_ arterial oxygen tension, *PaCO*_*2*_ arterial carbon dioxide tension, *SpO*_*2*_ oxygen saturation, *FiO*_*2*_ fraction of inspired oxygen, *CT* computed tomography, *CPAP* continuous positive airway pressure

**Fig. 1:**
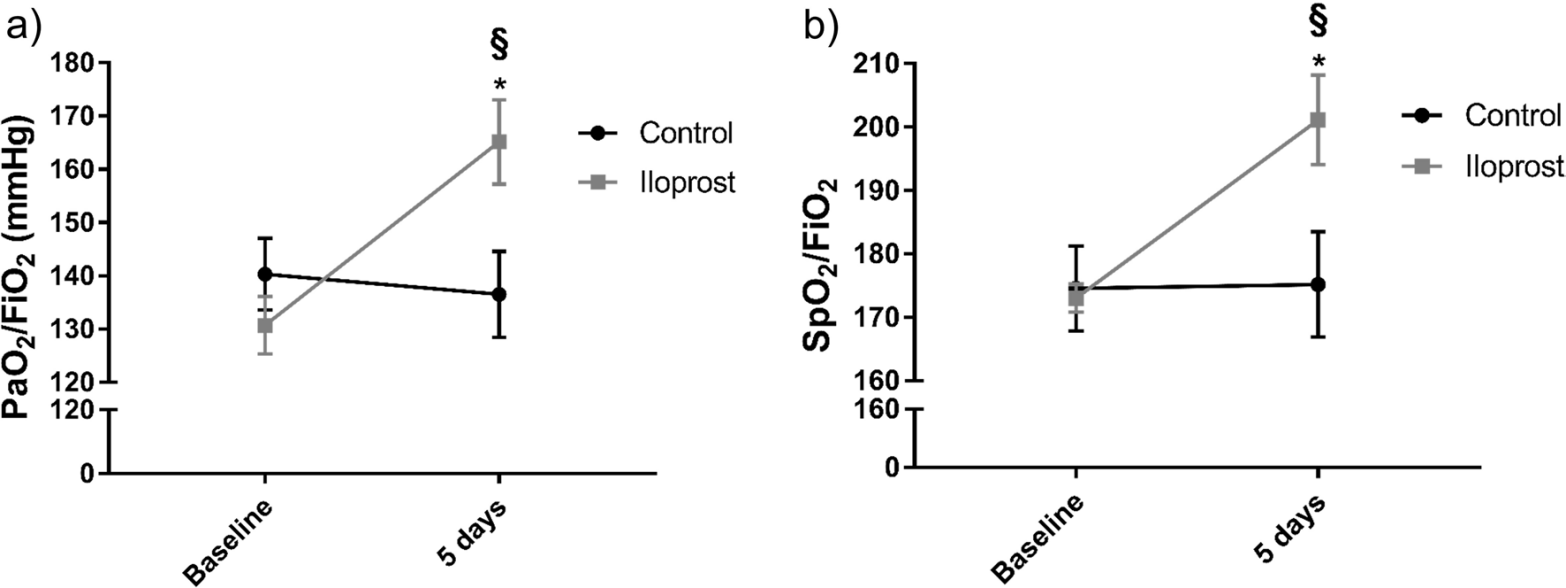
Effects of inhaled iloprost on oxygenation parameters **a** PaO_2_/FiO_2_;** b** SpO_2_/FiO_2_). Results are presented as mean ± SEM (*n* = 16–23). *PaO*_*2*_*/FiO*_*2*_ arterial oxygen tension-to-inspired oxygen fraction ratio, *SpO*_*2*_*/FiO*_*2*_ arterial oxygen saturation-to-inspired oxygen fraction ratio. Variables were compared with two-way ANOVA with Sidak’s multiple comparisons test. *p < 0.05. *Baseline versus 5 days; ^§^Control versus iloprost

In the context of COVID-19, still limited literature sources highlighted the usage of iloprost as potential therapeutic option [2, 3]. In the line with the literature, our findings revealed promising effects of inhaled iloprost with improved oxygenation parameters in patients with COVID-19-associated ARDS. It must be noted that our small pilot study is hypothesis generating rather than confirmatory and its results should be proved in randomized controlled trials.

## Data Availability

Data and materials can be obtained from the corresponding author upon the reasonable request.

## Abbreviations

ARDS: Acute respiratory distress syndrome
ICU: Intensive care unit
PaO_2_: Partial pressure of oxygen
FiO_2_: Fraction of inspired oxygen
SpO_2_: Oxygen saturation
NEWS-2: National Early Warning Score
AE: Adverse events
